# Alpha-1B Glycoprotein Is a Novel Hepatocyte-Derived Host Factor Associated with In Vitro Inhibition of HBV Replication and Hepatocellular Carcinoma Progression

**DOI:** 10.3390/cancers18040662

**Published:** 2026-02-18

**Authors:** Juan Lyu, Takuto Nosaka, Yosuke Murata, Yu Akazawa, Tomoko Tanaka, Kazuto Takahashi, Tatsushi Naito, Masahiro Ohtani, Lihong Zhang, Yasunari Nakamoto

**Affiliations:** 1 Second Department of Internal Medicine, Faculty of Medical Sciences, University of Fukui, Fukui 910-1193, Japan; rmyy02284@usx.edu.cn (J.L.); yosukem@u-fukui.ac.jp (Y.M.); aka0124@u-fukui.ac.jp (Y.A.); kawakami@u-fukui.ac.jp (T.T.); tkazuto@u-fukui.ac.jp (K.T.); naitot@u-fukui.ac.jp (T.N.); mohtani@u-fukui.ac.jp (M.O.); 2 Department of Clinical Laboratory, Shaoxing People’s Hospital, Shaoxing 312000, China; zhanglh@usx.edu.cn

**Keywords:** A1BG, hepatitis B virus (HBV), hepatocellular carcinoma (HCC), host factor, prognosis

## Abstract

Background: Chronic hepatitis B virus (HBV) infection is a major risk factor of hepatocellular carcinoma (HCC), and hepatocyte-derived host factors play important roles in HBV-associated tumor progression. Alpha-1B glycoprotein (A1BG) is a plasma glycoprotein reported to be dysregulated in multiple cancers. In this study, we investigated the functional role of A1BG in HBV-associated HCC progression. Methods: Both the HepG2 and HBV-transfected HepG2 cell lines were used to examine the biological effects of A1BG. A1BG expression was modulated using siRNA and a plasmid vector. A series of functional assays were conducted to assess cell proliferation, apoptosis, stemness, migration, and invasion. RNA microarray analysis and gene set enrichment analysis (GSEA) were performed to identify A1BG-regulated pathways. Results: Functionally, A1BG overexpression suppressed cell proliferation, stemness, migration, invasion, and HBV products while promoting apoptosis in both HepG2 and HBV-transfected HepG2 cells. In contrast, opposite effects were shown in the event of A1BG knockdown. Moreover, A1BG expression was reduced in HBV-associated HCC tissues and correlated with advanced pathological stage and poor prognosis. RNA microarray analysis and GSEA revealed the activation of anti-HBV-related genes and suppression of FGFR1 signaling and the matrix metalloproteinase pathway in A1BG-overexpressing cells. Conclusions: This study provides evidence that A1BG may be a novel host factor associated with the in vitro suppression of HBV replication and HCC progression by modulating pathways related to enhanced antiviral effects, reduced proliferative capacity and stemness, and suppression of EMT. These findings suggest that A1BG is a potential therapeutic target in HBV-related HCC.

## Simple Summary

Hepatitis B virus infection is a leading cause of liver cancer worldwide, yet the host factors involved in this process remain unclear. In our previous study, alpha-1B glycoprotein emerged as a candidate host factor linked to hepatitis B virus infection. As a result, in the present study, we further investigated its role in hepatitis B virus-associated liver cancer. Using liver cancer cell models transfected with hepatitis B virus and patient tissue data, we observed that reduced expression of this protein was associated with more aggressive tumor features and poorer prognosis. Increasing its expression decreased viral markers and inhibited the growth and migration of liver cancer cells. These findings suggest that alpha-1B glycoprotein may exert both antiviral and antitumor effects and could be a potential therapeutic target.

## 1. Introduction

Hepatocellular carcinoma (HCC) is the most prevalent form of primary liver cancer and the third leading cause of cancer-related death globally [1]. Hepatitis B virus (HBV) infection contributes to 50–80% of virus-associated HCC cases, especially in regions with high HBV prevalence such as East Asia and sub-Saharan Africa [2,3,4].

HBV promotes hepatocarcinogenesis through diverse mechanisms, including the integration of HBV DNA into the host chromosomal DNA [5,6,7], persistent expression of viral oncoproteins such as Hepatitis B virus X protein (HBx) [8], and dysregulation of critical oncogenic pathways and oxidative stress pathways [9,10,11]. Despite advances in understanding HBV virology and viral oncogenesis, the roles played by host factors in modulating HBV-induced tumorigenesis are becoming increasingly recognized but have not yet been fully elucidated [12].

Notably, in our previous work, the expression of alpha-1B glycoprotein (A1BG) was upregulated in HBV-infected primary human hepatocytes but reduced in HBV-transfected HepG2 cells upon signal transducer and activator of transcription 1 (STAT1) knockdown, suggesting the existence of a potential link between A1BG and host responses [13]. A1BG is a plasma glycoprotein with sequence similarity to the variable regions of some immunoglobulin superfamily members, yet its physiological function remains largely unknown [14]. Emerging evidence shows that A1BG is aberrantly expressed in multiple malignancies. In HCC, A1BG expression is markedly reduced in both HCC cell models and tumor specimens [15]. Consistently, proteomic studies have revealed that A1BG can serve as a diagnostic biomarker for distinguishing HCC from benign liver diseases [16]. Dysregulated A1BG expression has also been reported in other types of cancer, including pancreatic, cervical, and bladder cancers, where it was proposed as a potential biomarker for diagnosis or disease monitoring [17,18,19,20,21]. Notably, most previous studies have primarily focused on the association between A1BG expression levels and tumor progression, while its functional role in tumor biology remains insufficiently characterized. In particular, whether A1BG exerts tumor-suppressive, tumor-promoting, or context-dependent effects has not been clearly established. Moreover, the potential antiviral function of A1BG and its role in HBV-related HCC development and downstream signaling pathways remain largely unexplored.

In this study, we aimed to explore the role of A1BG in HBV-related HCC progression. Through the performance of bioinformatics analysis using a public dataset and functional experiments in HBV-transfected HepG2 cells, we examined the impact of A1BG in regulating antiviral effects, tumor cell proliferation, stemness, apoptosis, migration, and invasion. RNA microarray analysis was employed to identify downstream targets and molecular pathways regulated by A1BG.

## 2. Materials and Methods

### 2.1. Cell Lines and Cell Culture

The human liver cancer cell line HepG2 was obtained from the American Type Culture Collection (Manassas, VA, USA). HepG2 cells were selected as the primary experimental model because they are a well-characterized human hepatocellular carcinoma cell line widely used in HBV-related and tumor biology research. The derived cell lines HepG2.1-E10 (wild-type) and HepG2.D11 (mutant-type) were established via stable transfection with 1.3-mer HBV genotype C2 plasmids carrying either the wild-type genome (AB819618) or a double mutant (basal core promoter (BCP) A1762T/G1764A and precore G1896A; AB819615), respectively, as described previously [13]. All the cell lines were cultured in RPMI-1640 (Sigma-Aldrich, St. Louis, MO, USA) containing 10% fetal bovine serum (FBS), 2 mmol/L of L-glutamine, 1 μmol/L of sodium pyruvate, 0.1 mmol/L of nonessential amino acids, and 100 U/mL of penicillin/50 μg/mL of streptomycin (Gibco, Grand Island, NY, USA) at 37 °C with 5% CO_2_ [13].

### 2.2. Small Interfering RNA (siRNA) Transfection

Cells were transfected with 25 nM of siRNA using DharmaFECT4 transfection reagent (Horizon Discovery, Lafayette, CO, USA) following the manufacturer’s protocol. The siRNA constructs were obtained from the siGENOME SMARTpool Cherry-pick library (Horizon Discovery), including a siGENOME Human A1BG siRNA pool (catalog No. M-011191-01-0005, targeting A1BG, Entrez Gene ID: 1) and a siGENOME Nontargeting Control siRNA pool (catalog No. D-001206-13-05), which was used as a negative control.

### 2.3. Vector Transfection

Cytomegalovirus (CMV) promoter-driven plasmids for gene overexpression were designed and constructed by VectorBuilder (https://en.vectorbuilder.com/). The human A1BG expression vector (VectorBuilder ID: VB230816-1677zcx), encoding human A1BG (Transcript ID: NM_130786.4), was cloned into a CMV promoter-driven high-copy plasmid backbone containing a pUC origin of replication and an ampicillin resistance cassette for bacterial selection. The corresponding empty vector control (VectorBuilder ID: VB900124-3802vhh), containing an ORF stuffer sequence, was constructed using the same CMV promoter and plasmid backbone to ensure experimental consistency. All plasmids were transfected into cells using lipofectamine LTX reagent (Thermo Fisher Scientific, Tokyo, Japan) following the manufacturer’s protocol.

### 2.4. ELISA (Enzyme-Linked Immunosorbent Assay)

The quantitative determination of A1BG concentrations in cell supernatant was performed using a Human A1BG sandwich ELISA kit (Cat.KE00130; Proteintech, Tokyo, Japan) according to the manufacturer’s instructions. Cell supernatant samples were diluted 1: 1000 for the A1BG overexpression vector group and 1:10 for the si-nontarget, si-A1BG, and empty vector groups prior to the analysis.

### 2.5. CCK-8 (Cell Counting Kit-8) Assay

Cancer cell suspensions (1.2 × 10^4^/100 μL) were added to a 96-well plate and incubated at 37 °C in 5% CO_2_ for 24h. Then, the cells were transfected with siRNA or a vector plasmid. Cell viability was assessed at 48 h and 72 h after transfection using the CCK-8 assay (Dojindo, Kumamoto, Japan) following the manufacturer’s guidelines [13]. After incubating with CCK-8 reagent at 37 °C for 1 h, absorbance was recorded at 450 nm using a microplate reader. Cell viability was expressed as a percentage relative to the corresponding control group.

### 2.6. Carboxyfluorescein Succinimidyl Ester (CFSE) Assay

Cells were seeded on day 0 and siRNA was transfected on day 1. Forty-eight hours post-siRNA transfection, HepG2 cells were harvested. Cell proliferation was assessed using the CellTrace CFSE Cell Proliferation Kit (Cat No. C34554, Thermo Fisher Scientific). Cells were washed with PBS, resuspended at a concentration of 1 × 10^6^ cells/mL, and labeled with 5 μM of CFSE for 20 min in the dark. Labeling was quenched with 5 volumes of complete medium, followed by washing. After labeling, the cells were seeded and cultured under standard conditions. The CFSE fluorescence intensity was measured via flow cytometry (BD FACSCantoTM II, BD Biosciences, San Jose, CA, USA) on day 1 and day 3. The relative CFSE intensity was calculated as the CFSE fluorescence intensity at day 3 normalized to that at day 1.

### 2.7. Cell Apoptosis Assay

Cells were seeded on day 0 and siRNA or a plasmid vector was transfected on day 1. Seventy-two hours post-transfection, cell apoptosis was assessed using FITC Annexin V and propidium iodide (PI) staining (556547, BD Pharmingen™, San Diego, CA, USA), followed by flow cytometric analysis. Briefly, the cells were harvested, washed twice with PBS buffer, resuspended in a binding buffer, and incubated with Annexin V-FITC and PI for 15 min. Following incubation, 400 μL of binding buffer was added, and the cells were analyzed within 1 h using a BD FACSCantoTM II Flow Cytometer (BD Biosciences, USA).

### 2.8. Cell Migration and Invasion Assay

Cell migration and invasion ability were detected using Transwell chambers (pore size 8 μm, Cat. No. 3422, Corning, NY, USA) with or without Matrigel matrix coating (Cat. No. 356234, Corning, NY, USA). Cells were seeded on day 0, and siRNA or a plasmid vector was transfected on day 1. Forty-eight hours post-transfection, the cells were harvested and counted. A total of 8 × 10^4^ cells in 200 μL of serum-free medium were seeded into the upper chamber, while 600 μL of medium containing 20% FBS was added to the lower chamber. After 48 h of incubation at 37 °C, non-migrated or non-invaded cells on the upper side of the membrane were removed with a cotton swab. Cells on the lower surface were fixed and stained using the Diff-quick Stain Kit (Sysmex, Kobe, Japan) and quantified using Image J software (v1.54j; National Institutes of Health, Bethesda, MD, USA).

### 2.9. Sphere Formation Assay

Cells were transfected with siRNA or a vector for 48 h, after which they were harvested, counted, and seeded at equal numbers into ultra-low attachment plates to undergo sphere formation. Single-cell suspensions were cultured in DMEM/F12 (Gibco, Grand Island, NY, USA) serum-free medium supplemented with epidermal growth factor (20 ng/mL; Gibco), basic fibroblast growth factor (10 ng/mL; Gibco), and B27 supplement (1:100; Gibco); they were then plated in a 24-well ultra-low attachment surface culture plate (Corning, NY, USA) at a density of 2500 cells per well [22]. On day 8, whole-well images were captured using a BZ-X800 microscope (Keyence, Osaka, Japan), and spheres with a diameter greater than 50 μm were quantified using ImageJ software. 

### 2.10. RNA and DNA Extraction and cDNA Synthesis

Total RNA was extracted using the RNeasy Mini Kit (Cat. No, 74106, QIAGEN, Hilden, Germany), and cDNA synthesis was performed using a High-Capacity RNA-to-cDNA Kit (Applied Biosystems, Foster City, CA, USA). DNA was extracted from the HepG2.D11 and HepG2.1-E10 cells using the SMITEST EX-R&D DNA extraction kit (MBL) [13].

### 2.11. Quantitative Gene Expression Analysis

Quantitative real-time PCR was carried out using the TaqManTM Gene Expression Master Mix (Cat. No, 4369016, Applied Biosystems™) on a StepOne Plus Real-Time PCR System (Applied Biosystems). Gene expression levels were normalized to Glyceraldehyde-3-phosphate dehydrogenase (GAPDH), and relative expression was calculated using the 2^−ΔΔCt^ method. The primers used in this study are listed in Table 1.

### 2.12. Quantification of HBsAg

The hepatitis B surface antigen (HBsAg) levels in the supernatant were determined using the Lumipulse HBsAg-HQ immunoassay (Fujirebio Inc., Tokyo, Japan) [13,23] and normalized to the corresponding nontarget siRNA or empty vector control within each experiment, with the results presented as the relative levels.

### 2.13. Bioinformatics Analysis of A1BG in HCC

The expression of A1BG in HCC and adjacent normal tissues was analyzed using the TCGA-LIHC (https://portal.gdc.cancer.gov/, accessed on 11 September 2025) and GSE121248 (including 70 patients with chronic HBV-positive HCC) (http://www.ncbi.nlm.nih.gov/geo/, accessed on 17 February 2025) datasets. TCGA-LIHC data were accessed and processed using the TCGAbiolinks package (v2.34.1), and GEO datasets were accessed using the GEOquery package (v2.74.0). Differential expression of A1BG between the tumor and adjacent normal tissues, as well as among patients with different tumor stages, was assessed using R software (version 4.4.2). In the TCGA-LIHC cohort, A1BG expression was first compared between all tumor tissues and adjacent normal tissues. In addition, paired analyses were performed to assess the differences in A1BG expression between tumor samples and their matched adjacent normal tissues. For unpaired comparisons, the Wilcoxon rank-sum test was used. For paired tumor-adjacent analyses, the Wilcoxon signed-rank test was applied. Subsequently, HCC patients with HBV-related etiology were extracted based on the available risk factor information, and A1BG expression in HBV-associated tumor tissues was compared with that in adjacent normal tissues. The optimal cutoff value for stratifying patients into high- and low-A1BG-expression groups was determined using the survminer R package (v0.5.0), which identifies the threshold that best separates survival outcomes based on the log-rank test. Survival differences between the two groups in all HCC patients, as well as in the HBV-associated HCC subgroup, were evaluated using Kaplan–Meier analysis.

This study complied with all the applicable data usage regulations and involved only secondary analyses of publicly available datasets; therefore, no additional ethical approval was required.

### 2.14. Microarray Analysis

Microarray analysis were performed using the SurePrint G3 Human Gene Expression 8 × 60 K v3 Microarray (Agilent Technologies, Santa Clara, CA, USA), as described previously [13]. Raw signal intensities were extracted using Feature Extraction software (v12.0.3.1, Agilent Technologies) and normalized via quantile normalization using GeneSpring software (v14.9.1; Agilent Technologies) from Hokkaido System Science Co., Ltd. (Hokkaido, Japan). Probes flagged as “Detected” in at least one condition were retained, and differentially expressed genes were identified using a fold change ≥ 1.5 (|log2FC| ≥ 0.58). The raw microarray data have been deposited in the NCBI Gene Expression Omnibus (GEO) under accession number GSE314415 (https://www.ncbi.nlm.nih.gov/geo/query/acc.cgi?acc=GSE314415, accessed on 19 December 1025). Functional enrichment analysis was performed using gene set enrichment analysis (GSEA) software (version 4.3.2), with false discovery rate (FDR) q-values < 0.25 and adjusted *p*-values < 0.05 considered significant.

### 2.15. Quantification of HBV DNA and HBV RNA

The levels of HBV DNA and HBV RNA were quantified using real-time PCR, as previously described [13]. Transferrin receptor (TFRC) and GAPDH served as the endogenous controls. All primers and probes were purchased from Takara Bio (Shiga, Japan) and Applied Biosystems (Table 2). Reactions were performed on a StepOne Plus Real-Time PCR System (Applied Biosystems, USA).

### 2.16. Statistical Analysis

All flow cytometric data were analyzed using FlowJo software (version 10. 8. 1), and all experiments were repeated at least three times. The results were shown as mean ± standard deviation (SD). The normality of the data distribution was assessed using the Shapiro–Wilk test, and comparisons between the two groups were performed using Student’s *t*-test or the Wilcoxon rank-sum test, depending on the results of the normality test. To make comparisons between multiple groups, one-way analysis of variance (ANOVA) followed by Tukey’s post hoc test were used for normally distributed data, while the Kruskal–Wallis test followed by Dunn’s multiple-comparison test were applied for non-normally distributed data. Kaplan–Meier survival curves were compared using the log-rank test. Statistical analysis was performed using R software (version 4.4.2) or GraphPad Prism software (version 10.2.3, San Diego, CA, USA). A two-sided *p*-value < 0.05 was considered statistically significant.

## 3. Results

### 3.1. A1BG as a Potential Prognostic Biomarker in HCC Patients

**Figure 1:**
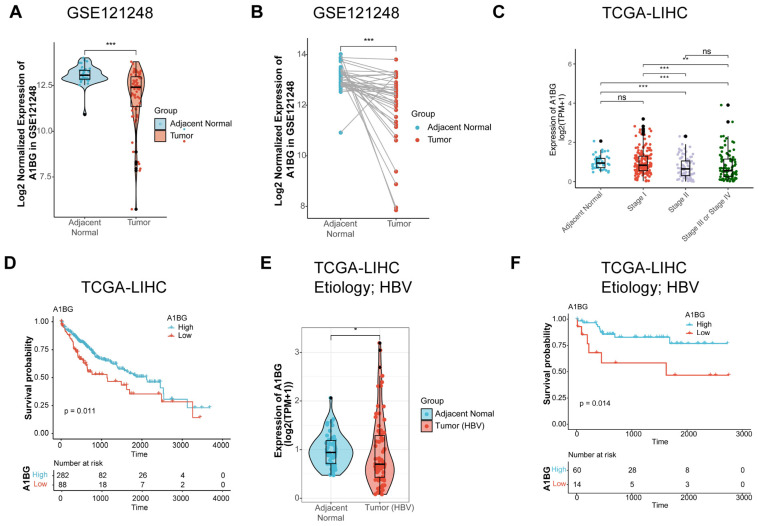
Bioinformatic analysis of A1BG expression in public datasets. (**A**,**B**) A1BG mRNA expression in adjacent normal and liver tumor tissues was assessed in the GSE121248 dataset. (**A**) Unpaired samples analysis; (**B**) paired samples analysis. (**C**) Correlation of A1BG expression with different tumor stages in the TCGA-LIHC dataset. (**D**) Kaplan–Meier overall survival analysis stratified by A1BG expression levels in the TCGA-LIHC dataset. (**E**) A1BG mRNA expression in tumor tissues from HBV-positive HCC patients compared with adjacent normal liver tissues in the TCGA-LIHC cohort. (**F**) Kaplan–Meier overall survival analysis of HBV-positive HCC patients in the TCGA-LIHC cohort stratified by A1BG expression level. In the violin and box plots, dots represent individual samples, the center line indicates the median, and the box represents the interquartile range. *** *p* < 0.001; ** *p* < 0.01; * *p* < 0.05; ns, not significant. Abbreviations: A1BG, alpha-1B glycoprotein; hepatocellular carcinoma (HCC).

To investigate the clinical relevance of A1BG in patients with HCC, we performed bioinformatic analysis using the GSE121248 and TCGA-LIHC and GSE121248 datasets. The expression of A1BG mRNA was lower in tumor tissues than in adjacent normal liver tissues, as demonstrated by both unpaired and paired sample analyses in the GSE121248 dataset (Figure 1A,B). In the TCGA-LIHC dataset, A1BG expression exhibited a stage-dependent decrease, with progressively reduced levels observed in advanced tumor stages (Figure 1C). Survival analysis further revealed that low A1BG expression was associated with poorer overall survival in HCC patients (Figure 1D); furthermore, when the analysis was restricted to HBV-positive HCC patients, a similar trend of reduced A1BG expression in tumor tissues was observed (Figure 1E). Importantly, the prognostic association was also observed in the HBV-positive subgroup, with patients that exhibited low A1BG expression showing significantly worse survival outcomes (Figure 1F). Collectively, these findings indicate that reduced A1BG expression is associated with HCC progression and poor prognosis, suggesting a potential tumor-suppressive relevance for A1BG in HCC.

### 3.2. Knockdown of A1BG Increased Cell Growth in HepG2 Cells

**Figure 2:**
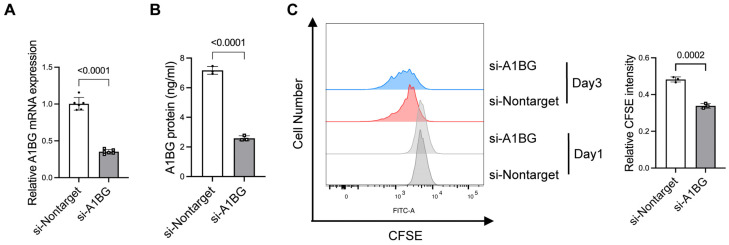
Effects of A1BG knockdown on cell proliferation in HepG2 cells. (**A**) Relative A1BG mRNA expression in HepG2 cells transfected with siA1BG, normalized to the si-nontarget control. (**B**) Secreted A1BG protein levels in culture supernatants measured using ELISA following siRNA transfection. (**C**) Cell proliferation assessed by CFSE dilution assay in siA1BG-transfected HepG2 cells. Relative CFSE intensity was calculated as the fluorescence intensity at day 3 normalized to that at day 1. Data were shown as mean ± SD (*n* = 3). Statistical significance was determined using Student’s *t*-test, and *p*-values were indicated. Abbreviations: siRNA, small interfering RNA; CFSE: carboxyfluorescein succinimidyl ester.

To elucidate the functional role of A1BG in liver cancer, we first assessed the effects of A1BG knockdown on cell proliferation in HepG2 cells. qRT-PCR and ELISA confirmed the efficient silencing of A1BG via siRNA at mRNA (Figure 2A) and protein levels (Figure 2B). The CFSE dilution assay revealed that A1BG knockdown promoted cell proliferation, as evidenced by the decrease in CFSE intensity (Figure 2C). Together, these findings suggest that A1BG knockdown promotes HepG2 cells’ proliferative capacity.

### 3.3. Overexpression of A1BG Inhibited Cell Growth and Motility in HepG2 Cells

**Figure 3:**
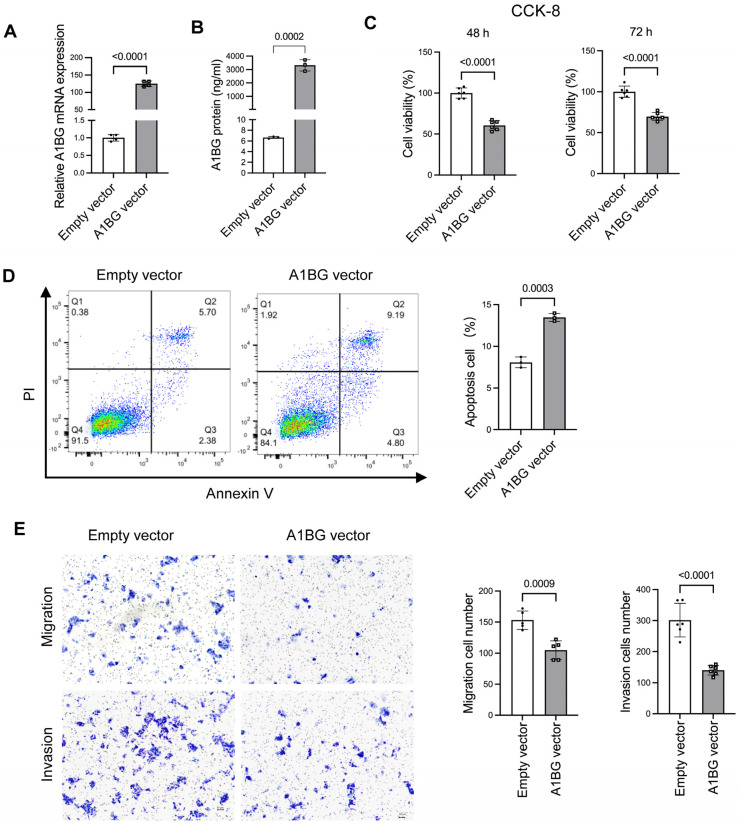
Effects of A1BG overexpression on cell viability, apoptosis, migration, and invasion in HepG2 cells. (**A**) A1BG mRNA levels in HepG2 cells transfected with A1BG vector and empty vector control, as determined using qRT-PCR. (**B**) Secreted A1BG protein levels in culture supernatants measured using ELISA following vector transfection. (**C**) Cell viability assessed using the CCK-8 assay at 48h and 72h post-transfection. (**D**) Cell apoptosis examined using Annexin V/PI staining followed by flow cytometry. Apoptotic cells were defined as the combined Annexin V^+^/PI^−^ (early apoptosis) and Annexin V^+^/PI^+^ (late apoptosis) populations. (**E**) Cell migration and invasion assays were performed using Transwell chambers in HepG2 cells transfected with empty vector and A1BG vector. Scale bar: 200 μm. Data were presented as relative levels normalized to the empty vector control group. Data were shown as mean ± SD. Statistical significance was determined using Student’s *t*-test, and *p*-values were indicated. Abbreviations: PI, propidium iodide.

To further investigate the functional role played by A1BG, we examined the effects of A1BG overexpression on cell proliferation, apoptosis, migration, and invasion in HepG2 cells. Transfection with an A1BG plasmid vector resulted in a marked increase in A1BG mRNA levels (Figure 3A) and secreted A1BG protein levels (Figure 3B). The CCK-8 assay showed that A1BG overexpression significantly reduced cell viability 48 h and 72 h post-transfection (Figure 3C). An increased proportion of apoptotic cells were observed in the A1BG-overexpressing group compared with the control group (Figure 3D). In addition, A1BG overexpression significantly reduced cell migration and invasion in HepG2 cells (Figure 3E). Collectively, these findings indicate that A1BG overexpression is associated with decreased cell viability, enhanced cell apoptosis, and reduced migratory and invasive behaviors in HepG2 cells.

### 3.4. Knockdown of A1BG Increased Tumor Progression and HBV Product Levels in HBV-Transfected HepG2 Cells

**Figure 4:**
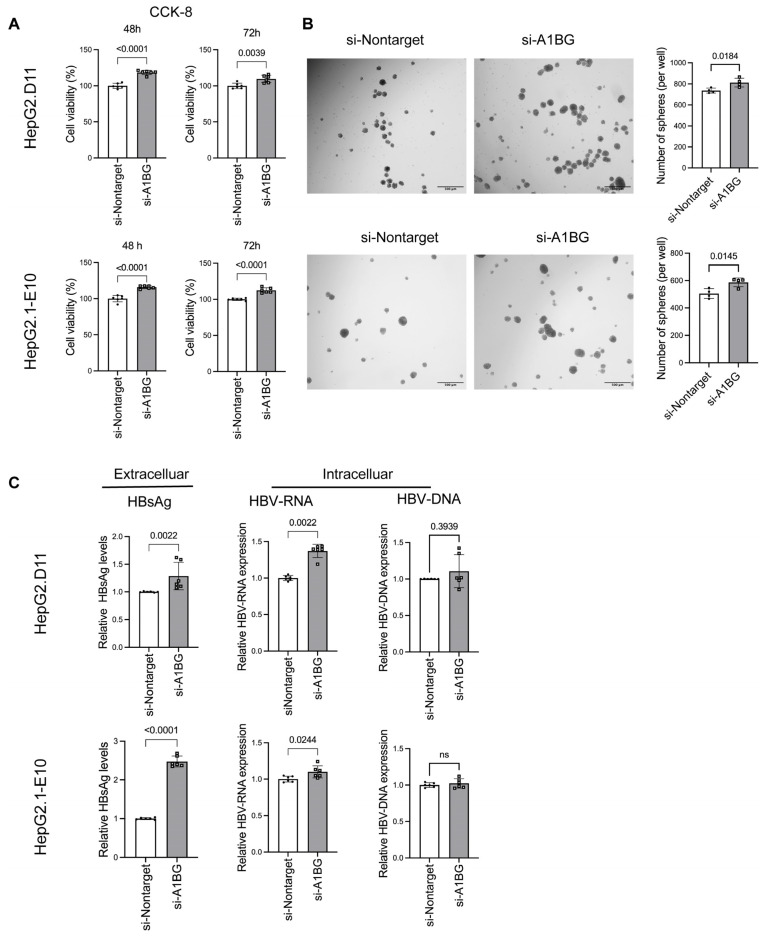
Knockdown of A1BG increased cell proliferation and sphere formation of HBV-transfected HepG2 cells. (**A**) Cell proliferation was assessed using the CCK-8 assay after siRNA transfection for 48 and 72 h (*n* = 6) in HepG2.D11 (mutant-type) and HepG2.1-E10 (wild-type) cells. (**B**) Sphere formation assays were performed to evaluate self-renewal capacity following A1BG knockdown in HepG2.D11 and HepG2.1-E10 cells (*n* = 4). Representative images and quantification of sphere numbers are shown. Scale bar: 500 μm. (**C**) Levels of HBV-related products, including extracellular HBsAg and intracellular HBV RNA and HBV DNA, were measured following A1BG knockdown in HepG2.D11 and HepG2.1-E10 (*n* = 6). Data are presented as relative levels normalized to the si-Nontarget control group. Data are shown as mean ± SD. Statistical significance was determined using Student’s *t*-test and *p*-values were indicated; ns, not significant. Abbreviations: CCK-8, cell counting kit-8; HBsAg, hepatitis B surface antigen.

To determine whether the effects of A1BG are consistent across different HBV genetic backgrounds, we performed parallel analyses in both wild type (HepG2.1-E10) and BCP/precore mutant (HepG2.D11) HBV-replicating HepG2-derived cell lines. Specifically, cell proliferation, sphere formation, and HBV-related markers were evaluated following A1BG knockdown in both models. The efficiency of A1BG knockdown in these two cell lines was validated at both the mRNA and protein levels using qRT-PCR (Figure S1A,C) and ELISA (Figure S1B,D), respectively. CCK-8 assays showed that A1BG knockdown significantly enhanced cell proliferation at both 48 and 72 h in both HepG2.D11 and HepG2.1-E10 cells (Figure 4A). Consistently, sphere formation assays demonstrated that A1BG knockdown increased the number of spheres formed in both HepG2.D11 and HepG2.1-E10 cells (Figure 4B). Moreover, knockdown of A1BG increased the levels of extracellular HBsAg and intracellular HBV-RNA (Figure 4C). Although intracellular HBV DNA levels did not reach statistical significance, a trend toward increased HBV DNA levels was observed compared with the control group (Figure 4C). Together, these results suggest that A1BG may negatively regulate cell growth and self-renewal capacity in HBV-transfected HepG2 cells and reduce HBV-related markers.

### 3.5. A1BG Overexpression Inhibited Cell Growth, Motility, and HBV Product Levels in HBV-Transfected HepG2 Cells

**Figure 5:**
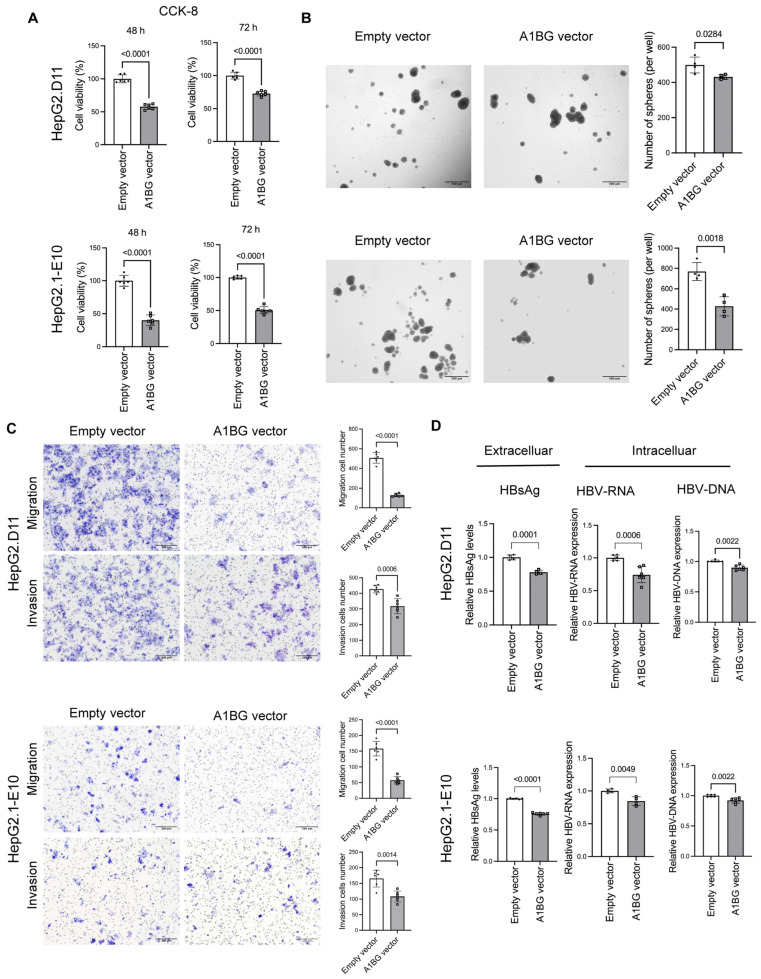
Effects of A1BG overexpression on cell proliferation, sphere formation, cell motility, and HBV-related products in HBV-transfected HepG2 cells. (**A**) Cell proliferation was assessed using CCK-8 assays at 48 and 72 h following A1BG overexpression in HepG2.D11 (mutant-type) and HepG2.1-E10 (wild-type) cells. (**B**) Sphere formation assays were performed to evaluate self-renewal capacity following A1BG overexpression in HepG2.D11 and HepG2.1-E10 cells (*n* = 4). Representative images and quantification of sphere numbers are shown. Scale bar: 500 μm. (**C**) Transwell assays were conducted to assess cell migration and invasion following A1BG overexpression in HepG2.D11 and HepG2.1-E10 cells (*n* = 6). Representative images and quantitative analyses are shown. Scale bar: 200 μm. (**D**) Levels of extracellular HBsAg, intracellular HBV RNA, and intracellular HBV DNA were measured following A1BG overexpression in HepG2.D11 and HepG2.1-E10 cells. Data were presented as relative levels normalized to the empty vector control group. Data were presented as mean ± SD from at least three independent experiments. Statistical significance was determined using Student’s *t*-test. *p*-values were indicated.

To further determine whether the effects of A1BG are consistent across different HBV genetic backgrounds, we performed parallel overexpression experiments in wild-type (HepG2.1-E10) and BCP/precore mutant (HepG2.D11) HBV-replicating HepG2-derived cell lines. Cell proliferation, sphere formation, and HBV-related markers were subsequently evaluated following A1BG overexpression in both models. The efficiency of A1BG overexpression in these two cell lines was validated at both the mRNA and protein levels using qRT-PCR (Figure S1E,G) and ELISA (Figure S1F,H), respectively. CCK-8 assays showed that A1BG overexpression significantly suppressed cell proliferation at both 48 and 72 h in HepG2.D11 and HepG2.1-E10 cells (Figure 5A). Consistently, sphere formation assays demonstrated that A1BG overexpression markedly reduced the number of spheres formed in both cell lines, indicating impaired self-renewal capacity (Figure 5B). In addition, Transwell assays revealed that A1BG overexpression significantly inhibited cell motility, as evidenced by decreased migration and invasion in HepG2.D11 and HepG2.1-E10 cells (Figure 5C). Regarding HBV-related products, A1BG overexpression resulted in reduced levels of extracellular HBsAg and intracellular HBV RNA and HBV DNA in both cell lines (Figure 5D). Together, these findings suggest that A1BG overexpression suppresses malignant cellular phenotypes in HBV-transfected HepG2 cells and is associated with a reduced level of HBV-related products.

### 3.6. RNA Microarray Analysis in Overexpression of A1BG in HepG2 Cells

**Figure 6:**
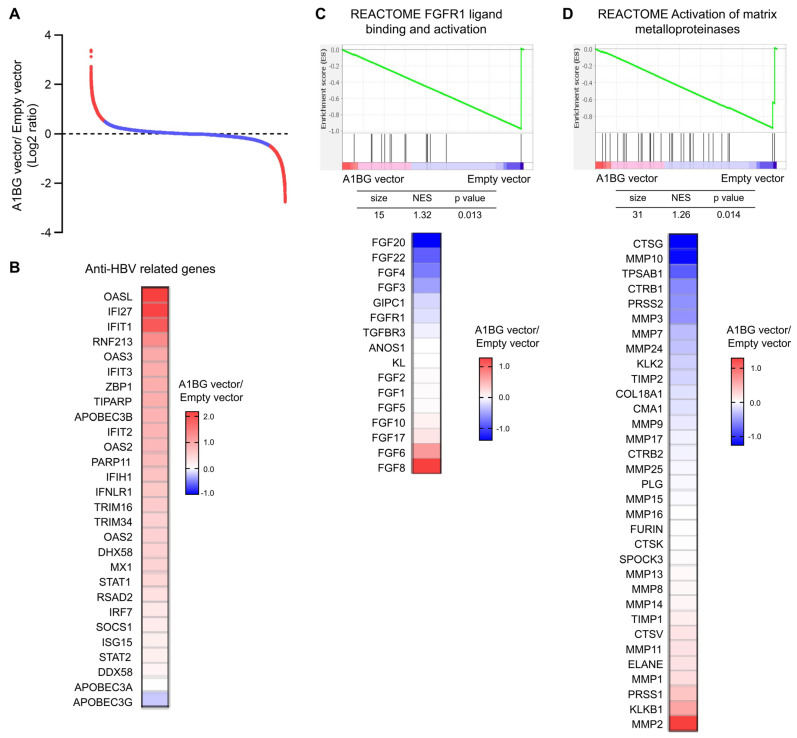
Identification of pathways regulated by A1BG using microarray analysis. (**A**) Microarray analysis of differentially expressed genes between HepG2 cells transfected with an A1BG expression vector and those transfected with an empty control vector at 72 h post-transfection. (**B**) Heatmap of the selected anti-HBV-related genes. (**C**,**D**) Gene set enrichment analysis (GSEA) identified significant suppression of FGFR1 ligand binding and activation signaling (**C**) and activation of MMP pathways (**D**) in A1BG-overexpressing cells. Heatmaps showed the expression patterns of genes associated with these pathways. Red and blue indicated relative upregulation and downregulation, respectively. Abbreviations: GSEA, gene set enrichment analysis; NES, normalized enrichment score; FGFR1, fibroblast growth factor receptor 1; MMP, matrix metalloproteinase.

To further investigate the biological functions of A1BG, we performed RNA microarray analysis to compare the gene expression profiles of A1BG-overexpressing HepG2 cells with control cells. A ranked gene expression plot illustrated the global distribution of differentially expressed genes (DEGs) between the two groups (Figure 6A). Notably, heatmap analysis of anti-HBV-related genes revealed that A1BG overexpression upregulated several interferon stimulated genes (ISGs), including interferon-induced proteins with tetratricopeptide repeats 1, 2, and 3 (IFIT1/2/3), members of the 2′-5′-oligoadenylate synthetase (OAS) family (including OAS2, OAS3, and OASL), myxovirus resistance protein 1 (MX1), radical S-adenosyl methionine domain-containing 2 (RSAD2, viperin), and ISG15, along with viral sensors such as interferon induced with helicase C domain 1 (IFIH1, also known as MDA5), DEAD-box helicase 58 (DDX58, also known as RIG-I), and DExH-box helicase 58 (DHX58, also known as LGP2) (Figure 6B). To further validate the involvement of the interferon signaling pathway, the qRT-PCR analysis showed that knockdown of A1BG resulted in the decreased expression of STAT1 and APOBEC3B (Figure S2A), supporting a positive association between A1BG and IFN–ISG axis activation.

Furthermore, GSEA revealed that the REACTOME FGFR1 (fibroblast growth factor receptor 1) ligand binding and activation pathway (NES = 1.32, *p* = 0.013) (Figure 6C) and the REACTOME activation of the matrix metalloproteinase (MMP) pathway (NES = 1.26, *p* = 0.014) (Figure 6D) were significantly enriched in control cells. Heatmap analysis of core enriched genes confirmed consistent expression patterns within each group (Figure 6C,D). Consistently, qRT-PCR validation demonstrated that A1BG overexpression downregulated the expression of FGF2 and MMP7 (Figure S2B), two representative genes involved in FGFR1 signaling and MMP-related pathways, respectively. Collectively, these results suggest that A1BG may be involved in both antiviral-associated responses and tumor-related processes in HepG2 cells, potentially through pathways linked to IFN–ISG signaling and FGFR1/MMPs-related programs.

## 4. Discussion

In this study, functional assays showed that A1BG suppressed cell proliferation, stemness, migration, invasion, and HBV products in HBV-transfected HepG2 cells. Moreover, A1BG expression was significantly reduced in HBV-associated HCC and correlated with advanced tumor stage and poor prognosis. Transcriptomic analysis revealed the enrichment of antiviral-related genes and suppression of FGFR1 and MMP signaling upon A1BG overexpression. These findings identify A1BG as a potential host factor associated with the suppression of HBV-associated HCC progression.

A1BG is a plasma glycoprotein with homology to immunoglobulin superfamily proteins, present in normal adult plasma at concentrations of approximately 22 mg/dL [14], yet its physiological function remains largely elusive. Previous proteomic and microarray studies have reported that A1BG expression is downregulated in HCC tissue, cell lines, and serum, proposing its potential utility as a biomarker to distinguish tumor tissues from non-tumor tissues [15,16]. Consistently, our bioinformatic analyses of public datasets confirmed that A1BG expression is significantly decreased in HBV-associated HCC tumor tissues compared with adjacent normal tissues. Moreover, reduced A1BG expression correlates with advanced tumor stage and poor prognosis, supporting the clinical relevance of A1BG as a potential prognostic biomarker in HCC.

Beyond its potential as a biomarker, our functional analyses further suggest a biological role of A1BG in tumor progression. Knockdown and overexpression experiments revealed that A1BG plays an important role in regulating tumor growth and motility. In HepG2 cells, overexpression of A1BG significantly increased cell apoptosis, whereas the knockdown of A1BG promoted cell proliferation. In HBV-transfected HepG2 cells, A1BG knockdown enhanced cell proliferation, sphere-forming ability, and HBV-related products (including extracellular HBsAg levels and intracellular HBV RNA and HBV DNA), whereas A1BG overexpression produced the opposite effects. Transwell assays showed that A1BG overexpression significantly suppressed the cell motility of HBV-transfected HepG2 cells. These effects were observed in both wild-type and BCP/precore mutant HBV-replicating cells. The BCP A1762T/G1764A and precore G1896A mutations are among the most frequently observed variants in chronic HBV infection [24,25,26,27]. The precore G1896A mutation introduces a premature stop codon that abolishes HBeAg production and has been associated with more advanced liver disease [28]. The BCP A1762T/G1764A mutation, located in the basal core promoter region, modulates viral transcription and has been independently linked to an increased risk of hepatocellular carcinoma [25,27,29]. Therefore, inclusion of this double mutant reflects a clinically relevant HBV genetic background. The consistent direction of A1BG effects in both wild-type and mutant models suggests that its functional impact is largely independent of this specific HBV mutation background under the conditions tested. Notably, baseline tumor-related phenotypes differed between the two HBV-replicating cell lines even in the absence of A1BG manipulation. These differences likely reflect intrinsic clonal variation resulting from independently established stable cell lines. Together, our findings indicate that A1BG may influence tumor progression in the context of HBV-replicating HCC models; however, whether this effect is HBV-dependent requires further investigation. The observed modulation of HBV-related products further suggests a potential role of A1BG in host–virus interactions; however, the underlying mechanisms require further investigation.

HBV is a partially double-stranded DNA virus with a complex lifecycle. A hallmark of HBV infection is its strong immune evasion capacity [30]. HBV persistence in hepatocytes is associated with attenuation of viral nucleic acid sensing, disruption of IFN signaling, and suppression of ISG induction [30,31,32]. Cytosolic RNA sensors, including DDX58, DHX58, and IFIH1, have been reported to act cooperatively in viral RNA sensing and initiation of antiviral responses [33,34,35,36]. In this study, we showed that A1BG upregulated the expression of IFIH1, DHX58, and DDX58, suggesting an enhanced capacity of hepatocytes to sense HBV-associated RNA intermediates. Consistent with this, components of the type III interferon signaling pathway, including IFNLR1 and the downstream transcription factors STAT1, STAT2, and IRF7, as well as multiple antiviral ISGs such as IFIT1/2/3, OAS2/3/OASL, MX1, RSAD2, and ISG15, were also upregulated. Previous studies have shown that activation of this pathway contributes to inhibition of HBV replication and viral protein expression [32,37,38,39]. Together, these findings suggest that A1BG may contribute to the restoration of antiviral signaling pathways that are suppressed during HBV infection.

In addition to anti-HBV-related genes, GSEA showed that FGFR1 ligand binding and activation signaling and MMP-associated pathways were significantly suppressed in A1BG-overexpressing HepG2 cells. Aberrant activation of FGFR1 signaling has been implicated in HCC progression by promoting tumor cell proliferation, differentiation, and survival [40]. MMPs are zinc-dependent endoproteases that regulate extracellular matrix degradation and remodeling and play important roles in HCC invasion, metastasis, and microenvironment remodeling [41]. Collectively, these findings indicate that A1BG may suppress tumor progression in HCC by attenuating growth factor-mediated signaling and extracellular matrix remodeling pathways.

Despite providing evidence for the involvement of A1BG in HCC progression and HBV replication in vitro, this study has several limitations. First, the HBV-replicating HepG2-derived cell models do not fully recapitulate all stages of the HBV lifecycle; further investigation into additional viral markers (e.g., HBeAg, HBV core protein, extracellular HBV DNA and HBV cccDNA) and infection-based models (e.g., NTCP-dependent systems or primary hepatocytes) is required. Second, the use of multiple independent siRNAs targeting different regions of A1BG, together with rescue experiments, would be necessary in future studies. Third, because cancer-related assays were mainly performed in HBV-replicating HepG2 models, the present data cannot distinguish between HBV-dependent and HBV-independent effects of A1BG. Future studies should include validation in HBV-negative HCC cells and additional HCC models, as well as experimental modulation of HBV status. Fourth, although the results of the transcriptomic analyses suggested associations with antiviral- and tumor-related pathways, comprehensive RNA-seq-based analyses and mechanistic validation will be necessary to delineate the molecular mechanisms. Fifth, although both WT and mutant HBV-replicating cell lines were included, mutation-specific effects should be examined in future studies. Finally, given that A1BG is a secreted plasma protein, future studies should investigate the relationship between serum A1BG levels and clinical features, as well as the potential of A1BG to be a biomarker for disease progression or a therapeutic target in HBV-associated HCC.

## 5. Conclusions

In conclusion, our findings reveal that A1BG is a novel host factor associated with the in vitro suppression of HBV replication and HCC progression by modulating pathways related to enhanced antiviral effects, reduced proliferative capacity and stemness, and suppression of EMT. These findings suggest that A1BG is a potential therapeutic target in HBV-associated HCC.

## Figures and Tables

**Table 1:** Primers used in this study.

Gene Symbol	Assay ID	Reporter	Quencher	Company
GAPDH	Hs02786624_g1	FAM	NFQ-MGB	Applied Biosystems
A1BG	Hs00373108_m1	FAM	NFQ-MGB	Applied Biosystems
STAT1	Hs01013996_m1	FAM	NFQ-MGB	Applied Biosystem
APOBEC3B	Hs00358981_m1	FAM	NFQ-MGB	Applied Biosystem
FGF2	Hs00266645_m1	FAM	NFQ-MGB	Applied Biosystem
MMP7	Hs01042796_m1	FAM	NFQ-MGB	Applied Biosystem

**Table 2:** Nucleotide sequences of primers and probe used for real-time quantitative PCR.

Primer Name		Nucleotide Sequences of Primers and Probe (5′-3′)	Nucleotide Position in HBV
HBV DNA, RNA-1816F	Forward	GCAACTTTTTCACCTCTGCCTA	1816−1837
HBV DNA, RNA-1974R	Reverse	GGAAAGAAGTCAGAAGGCAA	1974−1955
HBV DNA, RNA-1826F	Forward	CACCTCTGCCTAATCATC	1826−1843
HBV DNA, RNA-1947R	Reverse	AGTAACTCCACAGTAGCTCCAAATT	1947−1923
HBV DNA, RNA-Probe	Probe	(FAM)-TTCAAGCCTCCAAGCTGTGCCTTG-(TAMRA)	1863−1886
TFRC	Forward	GGACACCTATAAGGAACTGATTGAGA	
	Reverse	AGTCCAGGTTCAATTCAACATCATG	
	Probe	(FAM)-AATCACGAACTGACCAGCGACCTCTGC-(TAMRA)	

## Data Availability

The data of the current study are available from the corresponding author upon reasonable request.

## Supplementary Materials

The following supporting information can be downloaded at https://www.mdpi.com/article/10.3390/cancers18040662/s1, Figure S1: Validation of A1BG expression following knockdown and overexpression in HepG2.D11 and HepG2.1-E10 cells. (A–D) Validation of A1BG knockdown efficiency in HepG2.D11 (mutant-type) and HepG2.1-E10 (wild-type) cells. (A,C) Relative A1BG mRNA expression levels following siRNA-mediated knockdown (si-A1BG) compared with negative control siRNA (si-Nontarget), as determined by qRT-PCR. (B,D) A1BG protein levels after knockdown, quantified by ELISA. (E–H) Validation of A1BG overexpression efficiency in HepG2.D11 and HepG2.1-E10 cells. (E,G) Relative A1BG mRNA expression levels following overexpression (A1BG vector) compared with empty vector control. (F,H) A1BG protein levels after overexpression, measured by ELISA. Data are presented as mean ± SD from at least three independent experiments. Statistical significance was determined using Student’s *t*-test. *p*-values were indicated; Figure S2: Effects of A1BG modulation on antiviral and tumor related gene expression in HepG2 cells. (A) Relative mRNA expression levels of STAT1 and APOBEC3B after knockdown of A1BG. (B) Relative mRNA expression levels of FGF2 and MMP7 following A1BG overexpression. Gene expression levels were normalized to GAPDH. Data are presented as mean ± SD. Statistical significance was analyzed using Student’s *t*-test. * *p* < 0.05.

## Abbreviations

The following abbreviations are used in this manuscript:

A1BG	Alpha-1B glycoprotein
BCP	Basal core promoter
CFSE	Carboxyfluorescein succinimidyl ester
DEGs	Differentially expressed genes
DHX58	DExH-box helicase 58 (LGP2)
DDX58	DEAD-box helicase 58 (RIG-I)
EMT	Epithelial–mesenchymal transition
FGFR1	Fibroblast growth factor receptor 1
GSEA	Gene set enrichment analysis
HBsAg	Hepatitis B surface antigen
HCC	Hepatocellular carcinoma
IFIH1	Interferon induced with helicase C domain 1 (MDA5)
IFIT	Interferon-induced protein with tetratricopeptide repeats
IFN	Interferon
IFNLR1	Interferon lambda receptor 1
IRF7	Interferon regulatory factor 7
ISGs	Interferon stimulated genes
MMPs	Matrix metalloproteinases
MX1	Myxovirus resistance protein 1
OAS	2′-5′-Oligoadenylate synthetase
RSAD2	Radical S-adenosyl methionine domain-containing 2 (Viperin)
siRNA	Small interfering RNA
STAT	Signal transducer and activator of transcription
TCGA	The Cancer Genome Atlas
