# Supplementary material for: Alpha-1B Glycoprotein Is a Novel Hepatocyte-Derived Host Factor Associated with In Vitro Inhibition of HBV Replication and Hepatocellular Carcinoma Progression
Source: Cancers (Basel). 2026 Feb 18;18(4):662. doi: 10.3390/cancers18040662 (PMC12939741; doi:10.3390/cancers18040662)
Supplement: Supplementary file 1 [file cancers-18-00662-s001.zip › cancers-4114421-supplementary/Supplementary files/Supplementary Materials.docx]

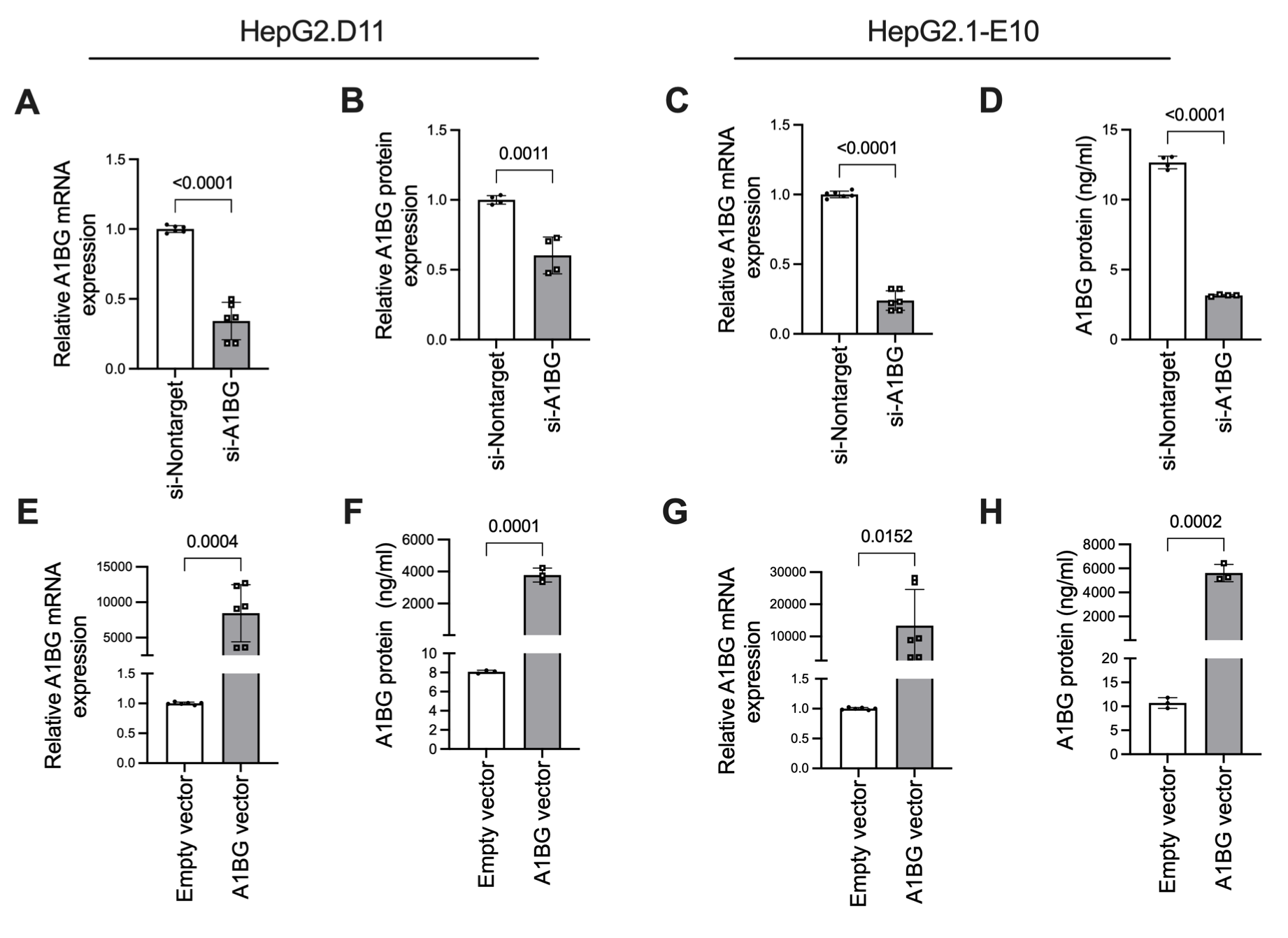


**Figure S1.** Validation of A1BG expression following knockdown and overexpression in HepG2.D11 and HepG2.1-E10 cells. (**A**–**D**) Validation of A1BG knockdown efficiency in HepG2.D11 (mutant-type) and HepG2.1-E10 (wild-type) cells. (**A**,**C**) Relative A1BG mRNA expression levels following siRNA-mediated knockdown (si-A1BG) compared with negative control siRNA (si-Nontarget), as determined by qRT-PCR. (**B**,**D**) A1BG protein levels after knockdown, quantified by ELISA. (**E**–**H**) Validation of A1BG overexpression efficiency in HepG2.D11 and HepG2.1-E10 cells. (**E**,**G**) Relative A1BG mRNA expression levels following overexpression (A1BG vector) compared with empty vector control. (**F**,**H**) A1BG protein levels after overexpression, measured by ELISA. Data are presented as mean ± SD from at least three independent experiments. Statistical significance was determined using Student’s t-test. *p*-values were indicated.


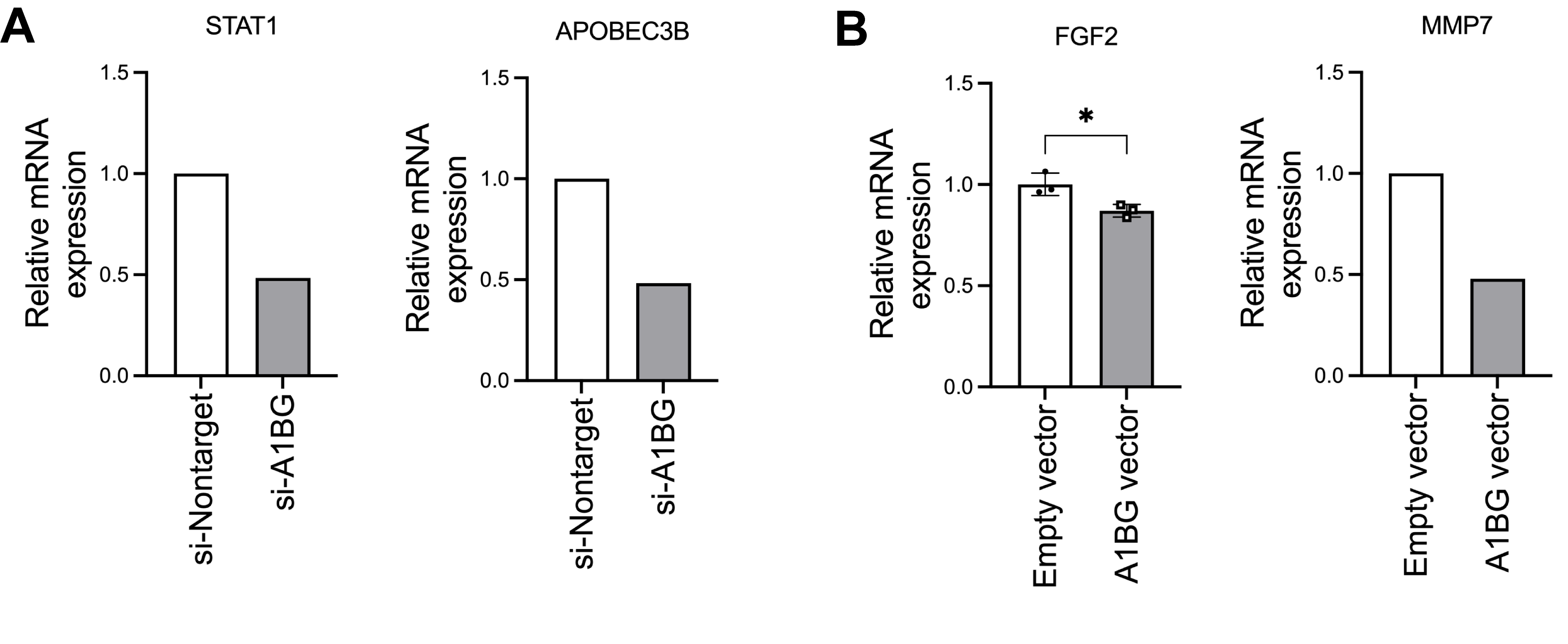


**Figure S2**. Effects of A1BG modulation on antiviral and tumor related gene expression in HepG2 cells. (**A**) Relative mRNA expression levels of STAT1 and APOBEC3B after knockdown of A1BG. (**B**) Relative mRNA expression levels of FGF2 and MMP7 following A1BG overexpression. Gene expression levels were normalized to GAPDH. Data are presented as mean ± SD. Statistical significance was analyzed using Student’s t-test. * *p* < 0.05.
