# Supplementary figures and images for: Alpha-1B Glycoprotein Is a Novel Hepatocyte-Derived Host Factor Associated with In Vitro Inhibition of HBV Replication and Hepatocellular Carcinoma Progression
Source: Cancers (Basel). 2026 Feb 18;18(4):662. doi: 10.3390/cancers18040662 (PMC12939741; doi:10.3390/cancers18040662)

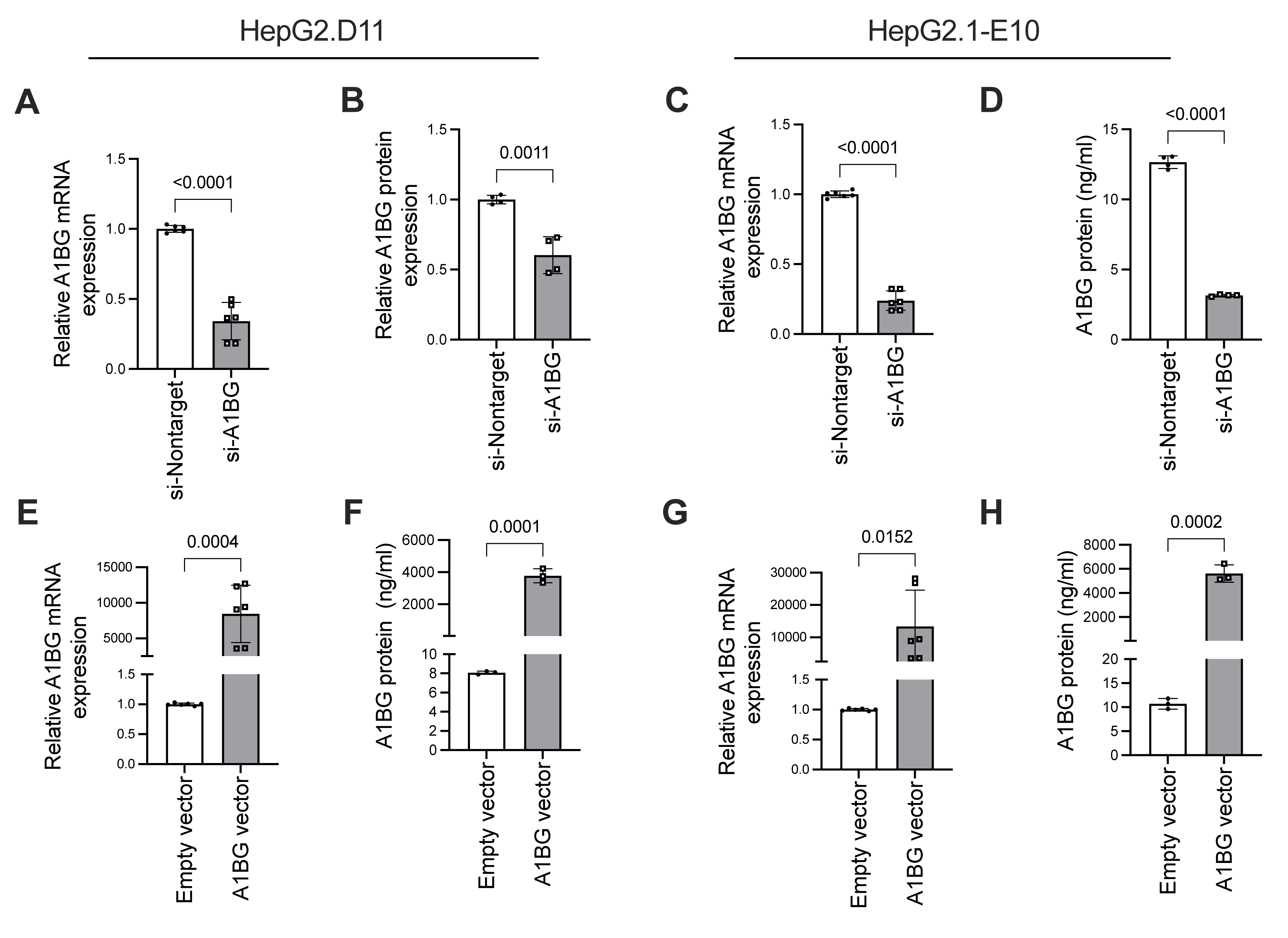

Supplement: Supplementary file 1 [file cancers-18-00662-s001.zip › cancers-4114421-supplementary/Supplementary files/Figure S1.tif]

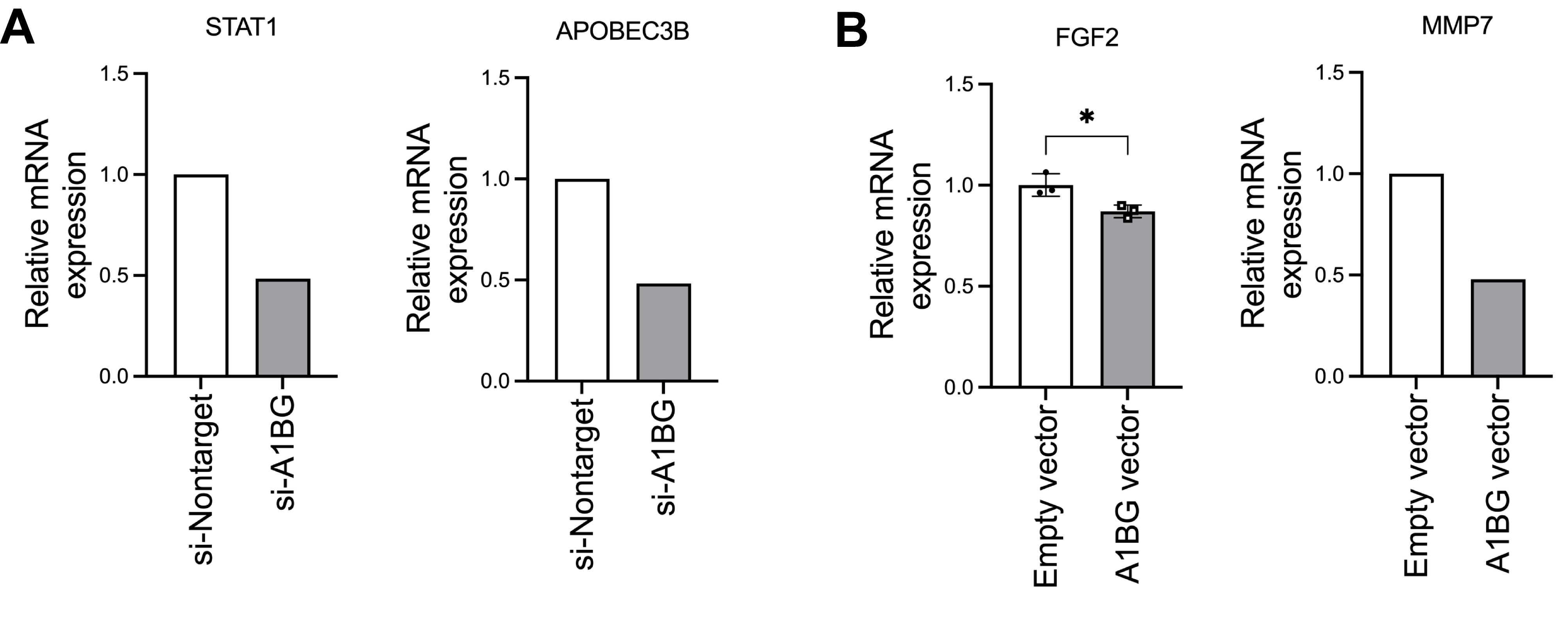

Supplement: Supplementary file 1 [file cancers-18-00662-s001.zip › cancers-4114421-supplementary/Supplementary files/Figure S2.tif]
